# Hepatitis Awareness Month and Testing Day, May 2018

**DOI:** 10.15585/mmwr.mm6719a1

**Published:** 2018-05-18

The United States commemorates National Hepatitis Awareness Month each May, and May 19 is designated as Hepatitis Testing Day. Viral hepatitis still persists as a major public health threat despite availability of preventive measures such as vaccines and therapies, including a curative treatment for hepatitis C virus (HCV) infection.

New cases of hepatitis B virus (HBV) and HCV infections are on the rise, largely among persons who inject drugs, with some attributed to the current U.S. opioid epidemic (*1*). Recent hepatitis A outbreaks have also occurred among unvaccinated injection drug users and homeless persons (*2*). Since August 2016, CDC has responded to hepatitis A outbreaks with high HBV/HCV co-infection, hospitalization, and mortality rates in multiple states (https://www.cdc.gov/hepatitis/outbreaks/2017March-HepatitisA.htm). New cases of perinatal HBV infection also continue (*1*); recently, CDC updated recommendations to strengthen vaccination among newborns and manage pregnant women (*3*).

This issue of *MMWR* includes an article about the promising outcomes of three HBV programs that implemented community-based services to improve HBV testing, linkage to care, and treatment among persons born in intermediate-high prevalence countries (*4*).

## References

[1] CDC. Viral hepatitis surveillance—United States, 2016. Atlanta, GA: US Department of Health and Human Services; 2016. https://www.cdc.gov/hepatitis/statistics/2016surveillance/pdfs/2016HepSurveillanceRpt.pdf.

[2] Williams WW, Lu PJ, O’Halloran A, Surveillance of vaccination coverage among adult populations—United States, 2015. MMWR Surveill Summ 2017;66(No. SS-11). 10.15585/mmwr.ss6611a1 28472027 PMC5829683

[3] Schillie S, Vellozzi C, Reingold A, Prevention of hepatitis B virus infection in the United States: recommendations of the Advisory Committee on Immunization Practices. MMWR Recomm Rep 2018;67(No. RR-1). 10.15585/mmwr.rr6701a1 PMC5837403 29939980

[4] Harris AM, Link-Gelles R, Kim K, Community-based services to improve testing and linkage to care among non–U.S.-born persons with chronic hepatitis B virus infection—three U.S. programs, October 2014– September 2017. MMWR Morb Mortal Wkly Rep 2018;67:541–6. 29771873 10.15585/mmwr.mm6719a2 PMC6048941

